# Loss of the Putative Catalytic Domain of HDAC4 Leads to Reduced Thermal Nociception and Seizures while Allowing Normal Bone Development

**DOI:** 10.1371/journal.pone.0006612

**Published:** 2009-08-12

**Authors:** Indrani Rajan, Katerina V. Savelieva, Gui-Lan Ye, Ching-yun Wang, Murtaza M. Malbari, Carl Friddle, Thomas H. Lanthorn, Wandong Zhang

**Affiliations:** Neuroscience Research, Lexicon Pharmaceuticals, Inc., The Woodlands, Texas, United States of America; L'université Pierre et Marie Curie, France

## Abstract

Histone deacetylase 4 (HDAC4) has been associated with muscle & bone development [1]–[6]. N-terminal MEF2 and RUNX2 binding domains of HDAC4 have been shown to mediate these effects *in vitro*. A complete gene knockout has been reported to result in premature ossification and associated defects resulting in postnatal lethality [6]. We report a viral insertion mutation that deletes the putative deacetylase domain, while preserving the N-terminal portion of the protein. Western blot and immuno-precipitation analysis confirm expression of truncated HDAC4 containing N-terminal amino acids 1-747. These mutant mice are viable, living to at least one year of age with no gross defects in muscle or bone. At 2–4 months of age no behavioral or physiological abnormalities were detected except for an increased latency to respond to a thermal nociceptive stimulus. As the mutant mice aged past 5 months, convulsions appeared, often elicited by handling. Our findings confirm the sufficiency of the N-terminal domain for muscle and bone development, while revealing other roles of HDAC4.

## Introduction

HDAC4 (histone deacetylase 4) is a member of the class IIa histone deacetylase family, which also includes HDAC5, HDAC7 and HDAC9. Class IIa histone deacetylases are defined [7] by having an extended N-terminal domain that interacts with specific transcription factors [4], [6], [8] in addition to a C-terminal deacetylase domain [9]–[11]. This class also contains cell signal responsive elements that affect nucleo-cytoplasmic shuttling [12]–[14] as well as cell-type restricted expression [15]. HDAC4 has been extensively studied, *in vitro*, with regards to the functions of the various domains, their interactions, and the effect of cell signaling events on its nuclear import-export. Extensive deletion analysis [4], [6], [8] coupled with *in vitro* assays have established that amino acids (aa) 1–660 is sufficient to interact with MEF2, Runx2, HDAC1, 14-3-3, and also for phosphorylation and nuclear export [3], [12], [13], [16]–[19].

*In vivo*, HDAC4 in concert with RUNX2, has been shown to be critical for bone development. HDAC4 null animals [6] have premature ossification of endochondral bone due to chondrocyte hypertrophy during development and do not survive to weaning. On the other hand, over-expression of HDAC4 resulted in inhibition of chondrocyte hypertrophy with delayed ossification [6]. These effects mirror the phenotype seen in RUNX2 null animals where ossification is delayed, while over-expression leads to premature ossification [20]–[22].

More recently a conditional knockout approach adopted to create a MEF2C deficit exclusively in different skeletal elements identified a critical function of MEF2C along with HDAC4 in bone development beyond their known role in muscle development [1]. The effect of MEF2C deficit on bone formation is mediated through interactions with HDAC4 such that the precocious ossification in HDAC4 null animals is mostly rescued by a single allele deletion of MEF2C. Conversely the MEF2C +/− phenotype of delayed ossification is shown to be rescued by deletion of the repressing HDAC4 [1]. Thus HDAC4 repression of RUNX2 and MEF2C is critical for normal bone development.

Most in vitro studies have identified the N-terminal region as being sufficient for HDAC4 functions in transcription repression, phosphorylation and nucleocytoplasmic shuttling. In contrast, the function of the deacetylase domain of HDAC4 is less clear [9]–[11]. Recently it has been shown, *in vitro*, to have a 1000 fold lower activity than class I enzymes against conventional substrates [11]. Activity against novel substrate, trifluoroacetamide, has been reported, but evidence is lacking for a physiological endogenous substrate. However, the putative deacetylase domain of HDAC4 has been shown to bind and recruit class I deacetylases like HDAC3 that account for physiological enzymatic activity in the complex [10]. While the catalytic activity of the HDAC4 domain remains controversial, the domain itself is critical for recruiting HDAC3.

While the sufficiency of the N-terminal portion of HDAC4 for nucleo-cytoplasmic shuttling and transcription repression has been demonstrated in *in vitro* systems, it has not been demonstrated that the N-terminal portion alone is sufficient for these functions *in vivo*. In this report, we describe a mutant mouse line generated by viral insertion that disrupted the C-terminal deacetylase domain while preserving the N-terminal domain.

## Results

**Figure 1:**
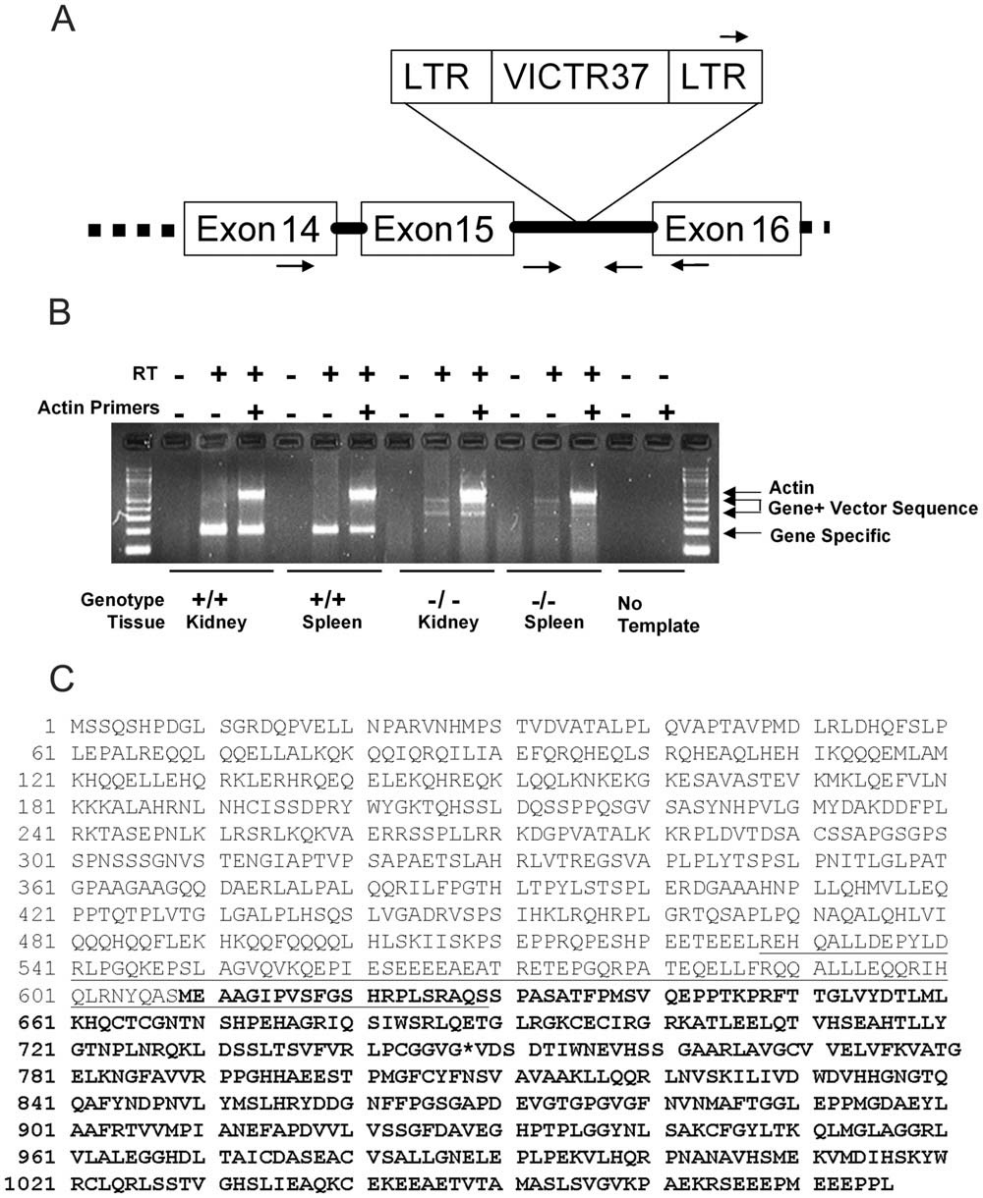
A. Diagram of the mutated HDAC4 locus. Genotyping and RT-PCR primers are indicated by arrows, as described in the methods. Primers in Exons 14 and 16 are used for RT-PCR to assay the endogenous transcript in wildtype and mutant mice. Primers in the LTR and intron (solid line) are used to genotype the wildtype and mutant mice. B. RT-PCR analysis of wildtype (+/+) and HDAC4^ΔC^ (−/−) mice. Kidney and spleen tissue was analyzed from one +/+ and one −/− mice. Each sample includes a negative control without Reverse Transcriptase, an assay with Hdac4 primers alone, and an assay that includes an internal positive control for Actin. C. Protein sequence of mouse HDAC4. Bold letters correspond to histone deacetylase (HDAC) domain. Underlined amino acid sequence (aa 528-629) is equivalent to the region of human HDAC4 antigen (human aa. 530-631) of Santa Cruz antibody. Asterisk * denotes the site of retroviral insertion, which would terminate protein translation and putatively produce a partial protein of 1-747aa with molecular weight of 83.3 kDa.

The mouse HDAC4 gene was mutated by gene trapping in our large-scale genome 5000™ knockout effort [23], [24]. A search of the OmniBank™ gene trap sequence database revealed several mutated ES cell clones with transcriptional sequence tags representing HDAC4. Five clones (OST37699, OST45739, OST68099, OST364354, and OST361114) were further characterized to determine the genomic location of the gene trap vector insertion and predict the extent of mutagenicity provided by each distinct gene trap mutation. Four of these clones were found to insert within the first intron of HDAC4 (NM_207225.1). These insertions occur prior to two possible alternate transcription start sites (TSS) for HDAC4 in the mouse, (alternate TSS 1 within intron 1 (representative EST BY272035) and alternate TSS 2 within intron 4 (representative mRNA AK029933)) therefore these mutations were not expected to disrupt expression of all isoforms of the HDAC4 gene. OST361114 was found to carry an insertion toward the end of the HDAC4 open reading frame, within intron 16 (Figure 1a). This gene trap mutation was selected for mouse production because it disrupted all transcript classes and occurred within the conserved histone deacetylase domain such that this domain's function should be effectively disrupted in the mutant animals. This mutation (HDAC4^ΔC^) resulted in viable pups born in the expected Mendelian ratio (Wt:169; Het:331; Hom HDAC4^ΔC^:146) that survived to adulthood and reproduced.

In light of the lack of generalized effects as compared to the complete KO [6], we hypothesized that the insertion strategy allowed for the expression of the N-terminal domain of the protein, thus preserving the domains necessary for overall development. RT-PCR analysis confirmed both the absence of full length HDAC4 transcript and the presence of a HDAC4-BGEO fusion transcript in the homozygous HDAC4^ΔC^ tissue (Figure 1b). Sequencing of the fusion transcript predicts a truncation of the HDAC4 protein after aa 747 (figure 1c).

**Figure 2:**
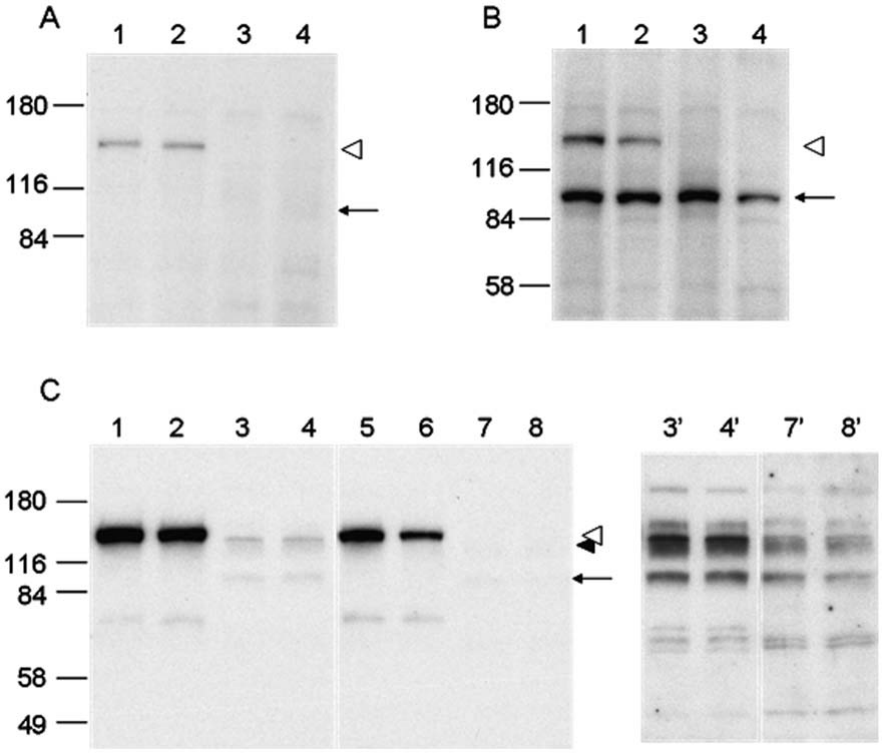
Western blot confirmation of partial HDAC4 protein in mutant animals. A: Western blot by anti-HDAC4 (Sigma, anti-aa1-19). B: Western blot by anti-HDAC4 (CST, anti-surrounding aa. 10). In both A and B, 1, 2 are wt brain and 3, 4 are HDAC4^ΔC^ brain. 1, 3 are cytosol fractions and 2, 4 are nuclear fractions. In both A and B, it is clear to see the full length HDAC4 of ∼140 kDa is absent in mutant brain labeled by open arrowhead. C: western blot by anti-HDAC4 (Santa Cruz, anti-aa528-629). 1, 2, 5, 6 are wt brain and 3, 4, 7, 8 are HDAC4^ΔC^ brain (3′, 4′ 7′ 8′ are longer exposure of 3, 4, 7, 8) and 1,2,3,4 are cytosol fractions and 5,6,7,8 are nuclear fractions. Arrow indicates the putative truncated protein of ∼95 kDa produced by viral insertion. The arrow in A marks the same size position but no truncated protein could be clearly seen. Arrow in B marks the same size position but there is a strong cross reacting band present in both wt and HDAC4^ΔC^, which block the possible revealing of the truncated protein in HDAC4^ΔC^ mutants. The arrowhead in C marked an irrelevant protein cross-reacted with HDAC4 antibody that is slightly smaller than full length HDAC4 and is present in both wild type and mutant.

**Figure 3:**
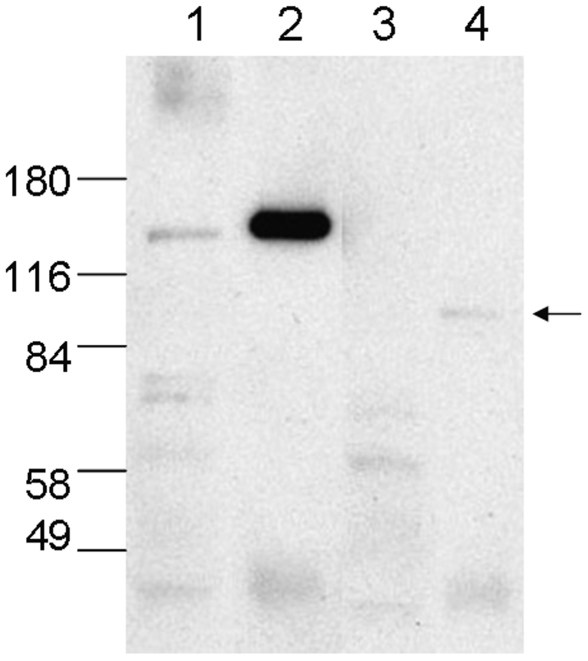
Immunoprecipitation by anti-HDAC4 antibody (Santa Cruz, anti-aa. 528-629) followed by western blot by anti-HDAC4 (Sigma monoclonal antibody, anti-aa. 1-19). 1, 2 are wts and 3, 4 are HDAC4^ΔC^ mutants. 1, 3 are inputs for IP and 2, 4 are IP products. The ∼95 kDa protein (arrow) was immunoprecipitated by Santa Cruz anti-HDAC4 and then was detected by the Sigma HDAC4 antibody in western blot. The fact that ∼95 kDa protein contained both recognition regions of two different HDAC4 antibodies further supported its HDAC4 origin.

Western analysis using several HDAC4 antibodies confirmed absence of the full length protein in brain tissue from HDAC4^ΔC^ animals (Figure 2). Additionally, an antibody (Ab) anti-aa528-629 identified a mutant-specific ∼95 kDa protein consistent with truncation of HDAC4, as the full length HDAC4 was about 140 kDa. Although expressed, this putative truncated protein is found at much reduced levels compared to the full length protein in wild type brain tissue. To further confirm this ∼95 kDa protein is indeed the HDAC4 product, we performed immuno-precipitation by anti-HDAC4 Ab. (anti-aa528-629) followed by western blot by anti-HDAC4 Ab. (anti-aa1-19). We found that the ∼95 kDa protein was precipitated by anti-aa528-629 Ab., and was recognized by anti-aa1-19 ab. (figure 3). The results confirm that a partial product, representing the N-terminal fragment, of HDAC4 is expressed in HDAC4^ΔC^ mice.

Further detailed phenotyping was performed (supplementary methods S1) to address the *in vivo* function of HDAC4 which had been precluded previously due to the early mortality of the complete null line [6]. HDAC4^ΔC^ mice appeared generally normal in terms of weight, length, as well as in a battery of physiological, metabolic, behavioral and histological assays at 8–14 weeks of age. A comprehensive phenotypic analysis (including a subset of behavioral tests derived from the Irwin screen) revealed no notable abnormalities across a wide range of behaviors as well as assays for cardiac, immune system, endocrine, and ophthalmic function (as an example, the phenotypic screen of VGLUT1 mice is accessible at: (http://www.informatics.jax.org/external/ko/lexicon/2383.html). At 12 months of age male, but not female, HDAC4^ΔC^ mice were smaller in size (WT 51.6±5.07 g, HDAC4^ΔC^ 34.04±2.8 g, P = 0.01, t-test).

HDAC4^ΔC^ mice did not show enlarged brains or exencephaly as has been reported previously for HDAC4 null mice [6]. Detailed histopathological analysis revealed no abnormalities in the HDAC4^ΔC^ brains (supplementary figure S1). In order to assess if the observed behavioral phenotype (below) was due to a subtle spinal cord abnormality or due to vertebral bone or muscle malformation, we carried out detailed histological analysis of the spinal cord, the vertebral column, and the vertebral muscles (supplementary figure S2). There were no differences noted in the HDAC4^ΔC^ animals compared to littermate controls.

**Figure 4:**
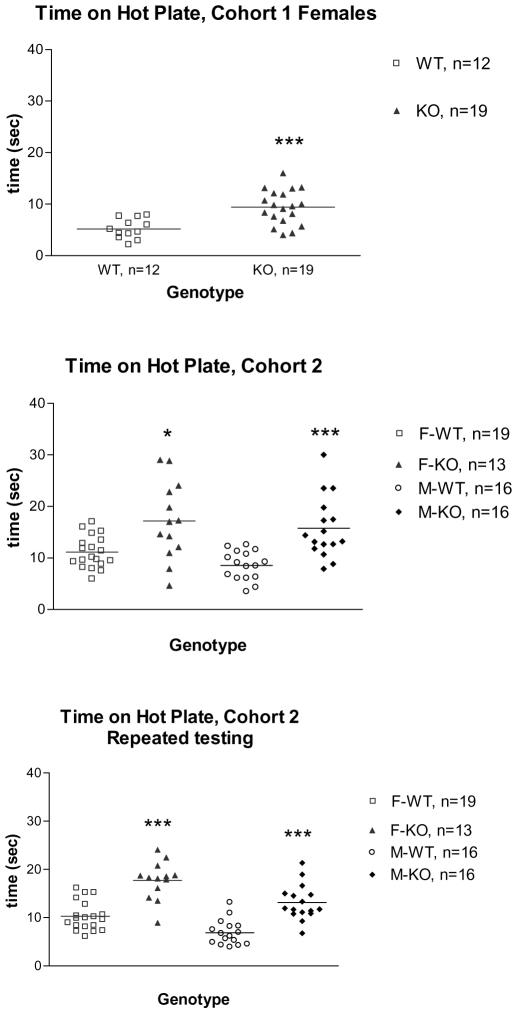
Latency to respond in Hot Plate assay. A. Latency to respond in females (cohort 1) was significantly higher in the HDAC4^ΔC^ mice, compared to WT littermate controls (t(29) = 4.0, *** −p<0.001 – unpaired two-tailed t-test). B. Latency to respond was significantly higher in the HDAC4^ΔC^ mice (cohort 2), compared to WT littermate controls (t(14) = 2.74, * −p<0.05 for females, and t(30) = 4.38, *** −p<0.001 for males– unpaired two-tailed t-test with Welch's correction). C. Latency to respond during repeated hot plate testing in the second cohort of mice. *** −p<0.0001 from WT littermates of the same sex (t(30) = 6.03 for females, and t(30) = 5.58 for males, unpaired two-tailed t-test).

Behavioral testing revealed specific phenotypic differences (Table 1). Increased latency to respond in the hot plate assay was evident and was observed in two different cohorts. This analgesic-like effect was noted in both sexes. In addition, subsequent hot plate testing on the second cohort of mice, one month after the initial test, confirmed and highlighted the persistence of the phenotype across age, test experience as well as generations (fig. 4). This effect was specific to an acute thermal nociceptive stimulus since there was no difference between genotypes in either phase of the formalin test (data not shown).

**Table 1:** Initial behavioral characterization of HDAC4^ΔC^ mice.

	WT	HDAC4^ΔC^
**Open Field**		
Total distance (cm)	2332±1150 (8)	2750±838 (8)
Center Time	293±148 (8)	279±100 (8)
Rearing	38±31 (8)	55±34 (8)
**Basal Body Temperature, °C**	35.9±0.5 (8)	35.5±0.7 (8)
**Stress-induced Hyperthermia**	1.3±0.7 (4)	1.65±0.65 (4)
**Inverted Screen**		
Fell down (ratio)	0/8	0/8
Climbed up (ratio)	4/8	8/8
**Marble Burying**	8.1±6.7 (8)	11.3±8.8 (8)
**Acoustic Startle Response**	395±189 (19)	457±269 (22)
**PPI (%)**		
pp4	19.5±28 (15)	25±28 (18)
pp8	25±29 (15)	34±26 (18)
pp12	51±17 (15)	47±23 (18)
pp20	74±11 (15)	72±18 (18)
**Tail suspension**		
Immobility (sec)	148±36 (8)	118±35 (8)
**Hot Plate**		
Latency (sec)	8.5±2.6 (8)	20.5±7.5 (8) **

Data are expressed as mean±SD (N). Statistical analysis was performed for all assays. * −p<0.05, ** −p<0.01 from WT littermates (unpaired t-test).

Some of the mice from cohort 2 (12/gender/genotype) were subsequently tested (at 5 months) in the open field assay (OF) to determine whether possible changes in activity levels could contribute to the effects observed in the hot plate test. The female HDAC4^ΔC^ mice traveled a greater distance in the OF than WT females (WT 1538±240.5 cm, HDAC4^ΔC^ 3349±464.7 cm, t(22) = 3.46, P<0.01 t-test); there was no difference in total distance between the males (WT 1665±183.6 cm, HDAC4^ΔC^ 2205±294.4 cm, t(22) = 1.56, P = 0.13) indicating that changes in activity do not confound the acute pain phenotype.

During this later hot-plate testing, a number of the HDAC4^ΔC^ mice (10/26) exhibited spontaneous seizures. Seizure-like activity was also observed in (2/11) control mice. Seizures also occurred in the home cage (supplementary data video S1, S2, S3 & S4), when the cage was opened, or after mice were handled (supplementary video S5). In most cases seizure activity was preceded by eye blinking and consisted of head tilt (up), eye closure, full body muscle spasms, followed by falling onto the side and continuing to spasm for 1–10 sec. Once these symptoms had subsided and animals righted themselves, they often showed residual ear and eye twitches (supplementary data video S2). Some of the HDAC4^ΔC^ mice also exhibited unilateral eye blinks and ear twitches in the absence of overt tonic-clonic seizures, suggesting additional minor seizure activity.

**Figure 5:**
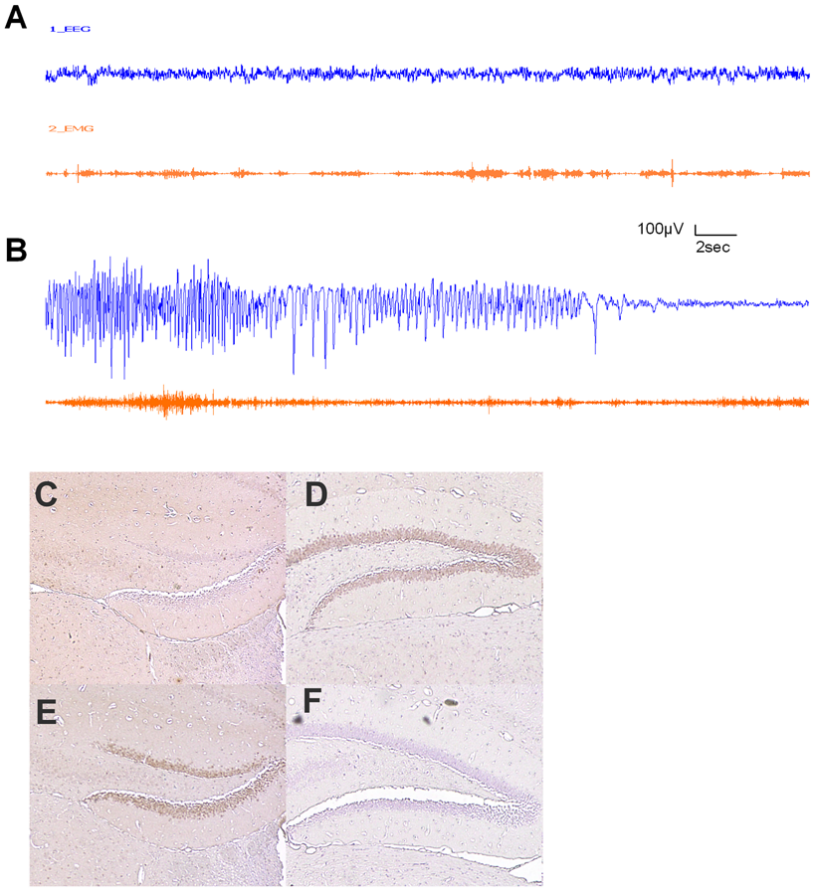
A &B: EEG/EMG recordings of a mutant mice exhibited behavioral convulsion after handling. (A) Continuous tracing of monopolar epidural EEG (upper trace) and trapezius EMG (lower trace) recording during waking. (B) shows generalized cortical discharge induced by gentle handling of the same mouse. The seizure activity is characterized by fast spiking concurrence with muscle potential during the tonic phase. The termination of the convulsion is characterized by EEG voltage depression after a slow spike transition. C–F: c-Fos expression in the hippocampus of WT and HDAC4^ΔC^ mice 60 minutes after handling and convulsions exhibited by the HDAC4^ΔC^. C) Very few c-Fos positive cells (brown) are seen in a HDAC4^ΔC^ devoid of seizures. D) Dentate gyrus (dg) neurons are positive for c-Fos in the HDAC4^ΔC^ animals expressing convulsions (D,E) but absent in the WT animal (F).

To more directly correlate the behavioral convulsions with neuronal activity, some of these mice underwent EEG/EMG telemetric monitoring before, during, and after handling. Two mutants in which behavioral convulsions had not been noted and four mutants with observed convulsions were tested along with five control littermates. During continuous monitoring, mice were taken out of the cage and gently handled. Two of the mutant mice with a previous history of convulsion exhibited it again on handling, while one without a history of behavioral convulsion, also exhibited generalized tonic seizure. None of the five WT mice tested exhibited abnormal EEG activity after handling. The pattern of epileptic EEG activity (fig. 5A, B) observed during convulsions was similar in all mice that exhibited behavioral convulsion. The electrographic seizure (fig. 5B) activity started with 10–20 sec of generalized cortical discharge with fast spiking, followed by a 20–30 sec period of slow spiking, and was terminated with 30–40 sec of EEG depression. Induction of c-Fos is an early marker of neuronal activity and is a robust marker of seizure activity [25]. c-Fos immunohistochemistry, performed one hour after handling, on brain sections of 5 animals of WT or HDAC4^ΔC^ revealed strong c-Fos expression in the dentate gyrus of HDAC4^ΔC^ animals that had exhibited convulsions in the preceding 60 minutes as compared to HDAC4^ΔC^ animals devoid of convulsions (fig. 5C, D, E) or control WT animals (fig. 5F). Overall, our EEG results suggest that seizures are exclusive to HDAC4^ΔC^ mice. However, since our initial observations noted convulsions in a few WT mice, and random instances of seizure activity in transgenic mice have been previously reported [26], it cannot be excluded that this behavior is magnified, rather than produced, by our genetic modification.

## Discussion

Our HDAC4^ΔC^ knockout phenotype indicates that the deacetylase domain of HDAC4 is dispensable for proper gross development of mice. In sharp contrast to the early mortality of the complete HDAC4 knockout mice [6], our C-terminal deletion resulted in viable mice that survived as long as we have observed them - over one year. *In vitro* transfection studies have shown that HDAC4 aa. 2-500 is sufficient to bind and repress Runx2 transcription activity [6] while Wang et al. [27] have demonstrated that HDAC4 118-208 is sufficient to interact with and repress MEF2C mediated transcription. Therefore it is highly likely that the expressed HDAC4 1-747 retains its binding to and repression of MEF2C and Runx2 *in vivo*, explaining the lack of chondrocyte hypertrophy in our mutant mice.

Extensive deletion analysis and *in vitro* transfection studies have also identified the minimum N-terminal protein domains of the HDAC4 protein sufficient to interact with binding proteins for nucleo-cytoplasmic trafficking, phosphorylation and recruitment of HDAC1 [4], [8], [12]–[14], [16]–[19]. *In vitro* assays have established that aa 119-208 is sufficient to interact with MEF2 and HDAC1 [4], [8]. While HDAC4 by itself lacks nuclear transport capabilities, evidence exists for MEF2 translocating HDAC4 to the nucleus where transcription repression is brought about by recruiting other HDAC's like HDAC1 [8]. Conversely phosphorylation of HDAC4 by calcium-calmodulin kinases (CAMKIV) [14], or CAMKII [16], or CAMKI [19] recruits the phospho-binding protein 14-3-3 and the HDAC4/14-3-3 complex is efficiently exported out of the nucleus [12], [13] preventing further repression. The phosphorylation sites of HDAC4 at Ser246, Ser467 and Ser632, and the 14-3-3 interaction site are also restricted to the N-terminal region such that a minimum of aa 1-660 is sufficient for HDAC4 to be trafficked in a nuclear-cytoplasmic manner [16]–[19]. Thus aa 1-747, retained in our HDAC4^ΔC^, encompasses all the necessary functional domains, for efficient nuclear import-export and nuclear repression, required for bone development and for overall gross development.

It is surprising that the low expression of HDAC4^ΔC^ protein had no observable impact on bone development. Arnold et al. [1] had observed that the precocious ossification in HDAC4 null animals is mostly rescued by a single allele deletion of MEF2C. Conversely the MEF2C +/− phenotype of delayed ossification has been shown to be rescued by deletion of the repressing HDAC4 suggesting an allele balance between HDAC4 and MEF2C capable of rescuing the bone phenotype. HDAC4^ΔC^ animals did not show reduced expression of MEF2C protein as analyzed by western blot (data not shown). Further quantitative analysis of protein expression in HDAC4^ΔC^ animals will have to be performed to address this question.

HDAC4^ΔC^ animals lack the histone deacetylase domain (HDAC domain) and the nuclear export signal. The integrity of the catalytic domain of HDAC4 is necessary for recruiting the HDAC3 N-CoR repressor complex and for subsequent deacetylase activity of the complex, but the deacetylase activity of HDAC4 is at best minimal [9], [11]. However, an *in vivo* function of the catalytic domain of type IIa HDAC's was confirmed by overexpression, in the mouse striatum, of HDAC5 transcripts in a cocaine reward model. The N-terminal domain was not able to mimic the attenuation in cocaine reward behavior observed after over-expression of full-length HDAC5. However, expression of the catalytic domain by itself replicated the effect shown by the full length protein [28]. Similar viral vector overexpression of full length HDAC4 in the striatum of mice led to reduced levels of histone H3 acetylation in the tissue and decreased the rewarding effects of cocaine in the place conditioning assay [29]. These *in vivo* results indicate a physiological requirement for this domain [28] and leads us to predict that HDAC4^ΔC^ mice would show hypersensitized responses in a chronic cocaine administration model.

It has been shown that the c-terminus of human HDAC4 contains a nuclear export activity [14]. If this were the only means for HDAC4 nuclear export, we would expect nuclear accumulation of the protein in HDAC4^ΔC^ mice. Nuclear accumulation of HDAC4 has been implicated in several pathways of regulation of neuronal cell death [30] and *in vitro* such nuclear accumulation has resulted in cerebellar granule neuron death. In the HDAC4^ΔC^ mice no neuronal cell loss is apparent in any of the brain regions analyzed (supplementary figure S1). In addition, fractionation assays of brain tissue showed HDAC4 in both nuclear and cytoplamic regions (figure 2) indicating that the c-terminal nuclear export signal is dispensable for appropriate nucleo-cytoplasmic trafficking of the protein.

HDAC4^ΔC^ mice were generally normal at 2–4 months of age except for a strong reduction in response to a thermal nociceptive stimulus. It is interesting to note that Runx1 knockout mice also show a similar analgesic-like effect [31], where Runx1 was shown to regulate expression of many nociceptive receptors or ion channels as well as the lamina specific innervation pattern of nociceptive afferents in the spinal cord. The Runx (runt-related protein) family of transcription factors play important roles in cell differentiation and tissue development. HDAC family members bind to Runx2 and act as transcription co-repressors in skeletal development [6], [32], [33]. Runx1 can recruit a number of class I and II HDACs as transcriptional co-repressors [34], [35] and is proposed to be a candidate interacting protein for HDAC4 based on its homology to Runx2/3 [36]. It is possible that HDAC4 together with Runx1 co-regulates expression of specific nociceptors. There was no alteration in acute chemical (formalin) induced nociception in our mutant, therefore we further hypothesize that HDAC4 regulates thermal nociceptors specifically, as has been described for Runx1. The normal responses to formalin-induced nociception also indicate that neuronal circuits for acute pain sensation and muscle reflexes for paw movement are not affected by the loss of the HDAC4 catalytic domain.

As the mice aged beyond 20 weeks the HDAC4^ΔC^ mice exhibited spontaneous convulsions that appeared to be exacerbated by handling. The factors leading to development of seizures are largely unknown. The HDAC4^ΔC^ mouse may serve as a novel genetic model to reveal the physiological and cellular mechanisms leading to epilepsy. It is possible that loss of the C-terminal deacetylase domain of HDAC4 leads to a dysfunction of synaptic transmission and neuronal excitability that underlie seizure. This seems somewhat at odds with the fact that the HDAC inhibitor, valproic acid [37], is a clinically effective anti-convulsant. It is not known if valproic acid acts on the novel enzymatic domain of HDAC4 *in vitro* or *in vivo*. Further study is required to reveal the interplay between HDAC4 and epilepsy.

In summary, our work confirms the N-terminal sufficiency of HDAC4 for transcriptional repression regulating skeletogenesis *in vivo*. HDAC4 has been implicated in neuronal function and survival [30], [38]–[40] and our work supports specific behavioral functions for HDAC4 *in vivo*. Although it is very possible that our behavioral phenotypes are a result of the loss of the putative deacetylase domain, they could also be due to the low expression of the N-terminal domain. Additional mutagenesis studies will be required to examine this.

## Materials and Methods

### Generation of HDAC4^ΔC^ animals

HDAC4^ΔC^ mice were generated at Lexicon Pharmaceuticals, by gene trapping in ES cells, identification of trapped genes by using OmniBank™ Sequence Tags (OSTs) and characterization of retroviral gene-trap vector insertion points as previously [23], [24]. OmniBank ES cell clone OST361114 was used to generate HDAC4^ΔC^ mice as described in PMID [23]. All mice were of mixed genetic background (129SvEvBrd and C57Bl/6J). RT-PCR confirmation of the mutation was performed on RNA extracted from kidney and spleen of both a homozygous mutant mouse and a wildtype littermate. Primers were from the fourteenth and sixteenth coding exons: 5′-CAC ACA CTC CTC TAC GGC ACA AAT C and 5′-ACC TTG AAG ACC AGC TCC ACT ACA CA. This yielded an RT-PCR product of 201 bp. Gene trap vector VICTR37 sometimes generates a splicing artifact. Sequence analysis of the resulting RT-PCR product shows a frameshift and truncation of the endogenous ORF. Mice were genotyped by PCR. The wildtype locus yields a PCR product of 309 bp using primers 5′-GTA AAG GCC TGT GTC TTT GAC TTC C and 5′-ACC CCA GGA TTC CAG GCT GCT ATC A. The mutant locus yields a PCR product of 166 bp using primers 5′-AAA TGG CGT TAC TTA AGC TAG CTT GC and 5′-ACC CCA GGA TTC CAG GCT GCT ATC A.

### Ethics Statement

Research related to use of mice was conducted under guideline of Lexicon Institutional Animal Care and Use Committee. All work was performed in accordance with Public Health Service policies and the Animal Welfare Act.

### Behavioral Testing

Only the assays in which significant differences were observed are described below (for descriptions of other assays see Supplementary Methods S1). Behavioral assays were performed on multiple cohorts of mice. Cohort -1 consisted of 12 female WT and 19 female HDAC4^ΔC^ tested in the HP and formalin assays; cohort-2 consisted of 16 male WT, 16 male HDAC4^ΔC^, 19 female WT, and 13 female HDAC4^ΔC^ tested in the open field, HP and formalin assays; Hot plate testing was performed twice on the second cohort of mice – prior to the formalin test and then again one month after the formalin test, to see if observed phenotype was still present after additional testing history.

### Locomotor activity

Locomotor and exploratory behaviors were recorded with twelve Digiscan open field (OF) apparatus and Versamax software (Accuscan Instruments, Inc., Columbus, OH). A large arena (42 cm×42 cm) with infrared beams at three different levels was used to record horizontal locomotor activity (total distance), vertical movement (rearing), and investigation into the 4 holes in the floor of the open-field (hole poke). Two florescent lamps positioned over each chamber provided light levels of 800 lux in the center of each open field arena. Each animal was placed in the center of the open field and its activity was measured for 20 minutes. The total distance traveled (cm), vertical movement number (rearing), number of hole pokes, time spent in the center of the OF (time-in-center), distance traveled in the center of the OF (center distance), and center/total distance ratio over the intervals were recorded using Versadat program (Accuscan Instruments). The OF center area measured 20×20 cm.

**Hot Plate test** to assess nociceptive response to an acute thermal pain stimulus was performed using a Columbus Instruments Hotplate Analgesia meter (model 1440). The hot plate was set to 55° C and controlled to within 0.1° C. The size of the heated surface area was 10″×10″×0.75″. The response time for each animal (hind-paw flinching) was automatically recorded by the computer when the experimenter blind to genotype of the animals tested pressed the stop button on the chamber.

### Biochemistry

HDAC4 antibodies were purchased from Santa Cruz (sc-11418, anti-aa. 528-629, rabbit), Cell Signaling Technology (#2072, anti-surrounding aa. 10, rabbit) and Sigma (anti-aa. 1-19, mouse monoclonal, clone HDAC-144). For tissue fractionation, mouse brains were dissected and homogenized in 0.32 M sucrose, 1 mM NaHCO3, 1× protease inhibitor, spun at 1400 g for 10minutes to obtain pellet (P1, nuclear fraction) and supernatant (S1, cytosol fraction). P1 was further extracted by RIPA buffer (10 mM Tris, 1 mM EDTA, 150 mM NaCl, 1% Triton, 1% DOC, 0.1% SDS) and spun at 18,000 g for 20 min to obtain supernatant (extracted P1). Protein concentration was determined and 20 micorgrams of protein were loaded to SDS-PAGE for western blotting. For immunoprecipitation the dissected mouse whole brain was directly homogenized in RIPA buffer, the insoluble material was removed by centrifugation and 2 milligrams of protein were used for immunoprecipitation. The antibodies were used for western or immunoprecipitation at dilution recommended by their suppliers.

### EEG/EMG Monitoring

Twelve male mice (6 months of age, 6 HDAC4^ΔC^ and 5 wts) were used for monitoring EEG/EMG before, during and after handling. Mice were surgically implanted with F-20-EET biopotential transmitters (DSI, Wisconsin US) as described by Weiergraber et al [41]. Briefly, mice were anesthetized with isoflurane. After the transmitter was place on top of the intestines, biopotential leads were directed caudally to a dorsal neck incision through a subcutaneous tunnel. The animal was then placed in a stereotaxic apparatus for cranial immobilization. The skull was perforated through the bone but before the dura membrane at 2 mm either side of the sagittal suture and 1 mm posterior to the bregma suture on left side and 1 mm anterior to the lambda suture on the right for ground and EEG leads implantation. EMG leads were secured in cervical trapezius muscles. Following 10–14 days of recovery from surgery and habituation, data were continuously acquired in EEG lab for 24 hours on a DATAQUEST ART 4.0 (Data Sciences International). EEG and EMG signals were amplified and filtered at 0.3–30 Hz for EEG and 30–300 Hz for EMG, digitized at a sampling rate of 250 Hz. After collection, EEG/EMG recordings were manually inspected for epileptic activity in aid of SLEEPSIGN software (KISSEI COMTEC CO.).

#### Pathology

Mice were anesthetized with Avertin. After incision of the vena cava inferior, mice were perfused with Harleco bouin fixation fluid (EMD chemicals, Inc., Gibbstown, NJ) or 4% paraformaldehyde through the left ventricle. Tissues were collected from HDAC4^ΔC^ mice and litter-mate control mice and were immersion fixed in 10% neutral buffered formalin. Bones were decalcified in 0.5 M EDTA pH 8.0 (Ambion, Austin, TX) for 7 days and all tissues were embedded in paraffin, sectioned at 4 µm and mounted on positively charged glass slides (Superfrost Plus, Fisher Scientific, Pittsburgh, PA) and stained with hematoxylin and eosin (H&E) for histopathologic examination. Several unstained sections both at 4 µm and 8 µm were collected on positively charged slides for future assays. The FD Rapid Timm stain kit (FD NeuroTechnologies, Inc., Baltimore, MD) was utilized on 8 µm sections according to the manufactures suggested protocol to compare neuropils in both the wild type and mutant mice. The Luxol Fast Blue kit (Poly Scientific, Bay Shore, NY) was used on 8 µm sections according to the manufactures suggested protocol to look for any differences in myelination in the wild type and mutant mice.

#### Immunohistochemistry

Formalin-fixed, paraffin-embedded tissue sections at 4 µm and mounted on positively charged glass slides (Superfrost Plus, Fisher Scientific, Pittsburgh, PA) were first deparaffinized in xylene and re-hydrated in PBS before incubating in the primary antibody. Commercially available c-Fos (Sigma-Aldrich, St. Louis, MO, cat # F7799) and MHC, slow and fast, (Novocastra Laboratories LTD., Newcastle, UK) were used to detect expression in the tissues of wild type and mutant mice. Mice intended for c-Fos expression analysis were collected 60 minutes after an episode of convulsion and prefused with 4% paraformaldehde and processed for paraffin embedding and sectioning. Both Myosin Heavy chain antibodies required antigen retrieval. Briefly, sections were immersed for 20 minutes in AR10 buffer, pH 10.0 (Biogenex, San Ramon, CA). After a 20 minute cool down the MHC slides were processed further. Endogenous peroxidase activity was blocked by incubation in 3.0% hydrogen peroxide in PBS for 5 min. Tissue sections were then blocked with 20% normal horse serum for 10 minutes and then primary antibodies (either 1∶25 mouse anti MHC slow, 1∶25 mouse anti MHC fast or 1∶500 Rabbit anti c-Fos) were applied for 1 hour at 37 degrees centigrade. After rinsing, sections were incubated for one hour in a 1∶500 dilution of the biotinylated secondary antibody (Horse anti-Mouse IgG, or Goat anti-Rabbit IgG, Vector, Burlingame, CA). Bound antibodies were detected by an avidin-biotin complex method (Elite ABC Kit, Vector) with 3,3′-diaminobenzidine as chromogenic substrate following the manufacturer's instructions. Negative controls included substitution of non-immune serum for the primary antibody and omission of the primary antibody. After counterstaining with hematoxylin, sections were mounted with Permount (Fisher Scientific, Pittsburgh, PA).

## Supporting Information

Methods S1 Behavioral assays
(0.03 MB DOC)

Figure S1 Histology of Brain: in each section left half is from WT and right half is from HDAC4^ΔC^ mice. A and C is Timm staining of a coronal section through the hippocampus of a WT and an HDAC4^ΔC^ mice. B, D, E and F is H&E staining of sections of a WT and a HDAC4^ΔC^ mice. B & D shows the hippocampus is similar in WT and a HDAC4^ΔC^ mice, E shows the cerebellar flocculus and F compares the spinal cord section of a WT and a HDAC4^ΔC^ mice.
(0.96 MB TIF)

Figure S2 Histology of vertebral muscles and bone; A, C & E is WT; B, D & F is HDAC4^ΔC^ mice. A&B shows IHC for MHC slow chain, C&D is IHC for MHC fast chain in lumbar vertebral muscles. E&F shows luxol fast blue stained lumbar vertebrae and spinal cord.
(1.55 MB TIF)

Video S1 Behavioral convulsions in the home cage. The videos sequentially record A) a cage of 5 male mice (3 mutant, 2 WT). White, black and one of the agouti mice are HDAC4 mutants and the black male mutant is convulsing. Notice that the animals are otherwise normal in body weight and general health.
(16.21 MB MPG)

Video S2 Behavioral convulsions in the home cage. Male mouse from video S1 recovering from convulsions: the mouse is exhibiting ear twitching before complete recovery.
(10.21 MB MPG)

Video S3 Behavioral convulsions in the home cage. C) A female black mutant mouse is exhibiting convulsions in a cage of 5 female mice (3 mutant, 2 WT) of which both of the black mice and one of the white mice are mutants. Notice that the animals are otherwise normal in body weight and general health.
(10.14 MB MPG)

Video S4 Behavioral convulsions in the home cage. D) Female mouse from Video S3 is recovering from convulsions. Notice that the animals are otherwise normal in body weight and general health.
(10.09 MB MPG)

Video S5 Handling induced seizure activity in HDAC4^ΔC^ mouse. The video shows a ∼20 week old female HDAC4 mutant mouse convulsing when placed in the open field apparatus.
(0.87 MB MPG)

## References

[1] Arnold MA, Kim Y, Czubryt MP, Phan D, McAnally J (2007). MEF2C transcription factor controls chondrocyte hypertrophy and bone development.. Dev Cell.

[2] Lu J, McKinsey TA, Zhang CL, Olson EN (2000). Regulation of skeletal myogenesis by association of the MEF2 transcription factor with class II histone deacetylases.. Mol Cell.

[3] McKinsey TA, Zhang CL, Lu J, Olson EN (2000). Signal-dependent nuclear export of a histone deacetylase regulates muscle differentiation.. Nature.

[4] Miska EA, Karlsson C, Langley E, Nielsen SJ, Pines J (1999). HDAC4 deacetylase associates with and represses the MEF2 transcription factor.. Embo J.

[5] Miska EA, Langley E, Wolf D, Karlsson C, Pines J (2001). Differential localization of HDAC4 orchestrates muscle differentiation.. Nucleic Acids Res.

[6] Vega RB, Matsuda K, Oh J, Barbosa AC, Yang X (2004). Histone deacetylase 4 controls chondrocyte hypertrophy during skeletogenesis.. Cell.

[7] Bertos NR, Wang AH, Yang XJ (2001). Class II histone deacetylases: structure, function, and regulation.. Biochem Cell Biol.

[8] Chan JK, Sun L, Yang XJ, Zhu G, Wu Z (2003). Functional characterization of an amino-terminal region of HDAC4 that possesses MEF2 binding and transcriptional repressive activity.. J Biol Chem.

[9] Bottomley MJ, Lo Surdo P, Di Giovine P, Cirillo A, Scarpelli R (2008). Structural and functional analysis of the human HDAC4 catalytic domain reveals a regulatory structural zinc-binding domain.. J Biol Chem.

[10] Fischle W, Dequiedt F, Hendzel MJ, Guenther MG, Lazar MA (2002). Enzymatic activity associated with class II HDACs is dependent on a multiprotein complex containing HDAC3 and SMRT/N-CoR.. Mol Cell.

[11] Lahm A, Paolini C, Pallaoro M, Nardi MC, Jones P (2007). Unraveling the hidden catalytic activity of vertebrate class IIa histone deacetylases.. Proc Natl Acad Sci U S A.

[12] McKinsey TA, Zhang CL, Olson EN (2001). Identification of a signal-responsive nuclear export sequence in class II histone deacetylases.. Mol Cell Biol.

[13] Wang AH, Yang XJ (2001). Histone deacetylase 4 possesses intrinsic nuclear import and export signals.. Mol Cell Biol.

[14] Zhao X, Ito A, Kane CD, Liao TS, Bolger TA (2001). The modular nature of histone deacetylase HDAC4 confers phosphorylation-dependent intracellular trafficking.. J Biol Chem.

[15] Verdin E, Dequiedt F, Kasler HG (2003). Class II histone deacetylases: versatile regulators.. Trends Genet.

[16] Backs J, Song K, Bezprozvannaya S, Chang S, Olson EN (2006). CaM kinase II selectively signals to histone deacetylase 4 during cardiomyocyte hypertrophy.. J Clin Invest.

[17] Grozinger CM, Schreiber SL (2000). Regulation of histone deacetylase 4 and 5 and transcriptional activity by 14-3-3-dependent cellular localization.. Proc Natl Acad Sci U S A.

[18] Wang AH, Kruhlak MJ, Wu J, Bertos NR, Vezmar M (2000). Regulation of histone deacetylase 4 by binding of 14-3-3 proteins.. Mol Cell Biol.

[19] McKinsey TA, Zhang CL, Olson EN (2000). Activation of the myocyte enhancer factor-2 transcription factor by calcium/calmodulin-dependent protein kinase-stimulated binding of 14-3-3 to histone deacetylase 5.. Proc Natl Acad Sci U S A.

[20] Hoshi K, Komori T, Ozawa H (1999). Morphological characterization of skeletal cells in Cbfa1-deficient mice.. Bone.

[21] Takeda S, Bonnamy JP, Owen MJ, Ducy P, Karsenty G (2001). Continuous expression of Cbfa1 in nonhypertrophic chondrocytes uncovers its ability to induce hypertrophic chondrocyte differentiation and partially rescues Cbfa1-deficient mice.. Genes Dev.

[22] Ueta C, Iwamoto M, Kanatani N, Yoshida C, Liu Y (2001). Skeletal malformations caused by overexpression of Cbfa1 or its dominant negative form in chondrocytes.. J Cell Biol.

[23] Zambrowicz BP, Abuin A, Ramirez-Solis R, Richter LJ, Piggott J (2003). Wnk1 kinase deficiency lowers blood pressure in mice: a gene-trap screen to identify potential targets for therapeutic intervention.. Proc Natl Acad Sci U S A.

[24] Zambrowicz BP, Friedrich GA, Buxton EC, Lilleberg SL, Person C (1998). Disruption and sequence identification of 2,000 genes in mouse embryonic stem cells.. Nature.

[25] Herrera DG, Robertson HA (1996). Activation of c-fos in the brain.. Prog Neurobiol.

[26] Baraban SC (2007). Emerging epilepsy models: insights from mice, flies, worms and fish.. Curr Opin Neurol.

[27] Wang AH, Bertos NR, Vezmar M, Pelletier N, Crosato M (1999). HDAC4, a human histone deacetylase related to yeast HDA1, is a transcriptional corepressor.. Mol Cell Biol.

[28] Renthal W, Maze I, Krishnan V, Covington HE, Xiao G (2007). Histone deacetylase 5 epigenetically controls behavioral adaptations to chronic emotional stimuli.. Neuron.

[29] Kumar A, Choi KH, Renthal W, Tsankova NM, Theobald DE (2005). Chromatin remodeling is a key mechanism underlying cocaine-induced plasticity in striatum.. Neuron.

[30] Bolger TA, Yao TP (2005). Intracellular trafficking of histone deacetylase 4 regulates neuronal cell death.. J Neurosci.

[31] Chen CL, Broom DC, Liu Y, de Nooij JC, Li Z (2006). Runx1 determines nociceptive sensory neuron phenotype and is required for thermal and neuropathic pain.. Neuron.

[32] Komori T (2005). Regulation of skeletal development by the Runx family of transcription factors.. J Cell Biochem.

[33] Schroeder TM, Kahler RA, Li X, Westendorf JJ (2004). Histone deacetylase 3 interacts with runx2 to repress the osteocalcin promoter and regulate osteoblast differentiation.. J Biol Chem.

[34] Durst KL, Hiebert SW (2004). Role of RUNX family members in transcriptional repression and gene silencing.. Oncogene.

[35] Reed-Inderbitzin E, Moreno-Miralles I, Vanden-Eynden SK, Xie J, Lutterbach B (2006). RUNX1 associates with histone deacetylases and SUV39H1 to repress transcription.. Oncogene.

[36] Hug BA (2004). HDAC4: a corepressor controlling bone development.. Cell.

[37] Khan N, Jeffers M, Kumar S, Hackett C, Boldog F (2008). Determination of the class and isoform selectivity of small-molecule histone deacetylase inhibitors.. Biochem J.

[38] Chawla S, Vanhoutte P, Arnold FJ, Huang CL, Bading H (2003). Neuronal activity-dependent nucleocytoplasmic shuttling of HDAC4 and HDAC5.. J Neurochem.

[39] Chen B, Cepko CL (2009). HDAC4 regulates neuronal survival in normal and diseased retinas.. Science.

[40] Majdzadeh N, Wang L, Morrison BE, Bassel-Duby R, Olson EN (2008). HDAC4 inhibits cell-cycle progression and protects neurons from cell death.. Dev Neurobiol.

[41] Weiergraber M, Henry M, Hescheler J, Smyth N, Schneider T (2005). Electrocorticographic and deep intracerebral EEG recording in mice using a telemetry system.. Brain Res Brain Res Protoc.

[42] Martin JR, Bos M, Jenck F, Moreau J, Mutel V (1998). 5-HT2C receptor agonists: pharmacological characteristics and therapeutic potential.. J Pharmacol Exp Ther.

