# Supplementary material for: Loss of the Putative Catalytic Domain of HDAC4 Leads to Reduced Thermal Nociception and Seizures while Allowing Normal Bone Development
Source: PLoS One. 2009 Aug 12;4(8):e6612. doi: 10.1371/journal.pone.0006612 (PMC2720538; doi:10.1371/journal.pone.0006612)
Supplement: Methods S1 — Behavioral assays (0.03 MB DOC) [file pone.0006612.s001.doc]

**Supplementary Methods**

**Behavioral assays:**

# Formalin Paw

Mice were tested for nociception with Automatic Nociception Analyzers (purchased from the Ozaki lab at University of California, San Diego). A metal band was placed around the left hind paw of each mouse with superglue 30 minutes prior to testing. After the 30-minute acclimation period, 20 µl of 5% formalin was subcutaneously injected in the dorsal surface of the left hind paw. Mice were individually housed in cylindrical chambers for 45 minutes. A computer recorded flinches per minute, total flinches for Phase I (acute phase = first 8 minutes), and total flinches for Phase II (tonic phase between 20 - 40 minutes) through an electromagnetic field.

*Stress-Induced Hyperthermia (SIH) and Basal Body Temperature*

Body temperature measurements were recorded with a Physitemp Instruments TH-5 Thermalert and RET-3 rectal probes. Mice were tested in their colony room to prevent additional stress from transporting cages. Basal body temperatures were recorded as T_1_. Ten minutes later the stress-enhanced body temperature was recorded and designated T_2_. The stress-induced hyperthermia is calculated as difference between T_2_ and T_1_.

*Inverted Screen*

For motor/strength coordination mice were tested with the inverted screen. Untrained mice were placed individually on top of a square (7.5 cm x 7.5 cm) wire screen, which was mounted horizontally on a metal rod. The rod was then rotated 180 degrees so that the mice were on the bottom of the screens. The following behavioral responses were recorded over a 1-minute testing session: fell off, did not climb, and climbed up. The results were expressed as a ratio.

*Marble burying test*

The marble burying test for anxiety-related and compulsive-like behaviors was performed as previously described [42]. Mice were individually placed into cages (24 x 18 x 13 cm) filled to a depth of 5 cm with clean bedding for a 30-minute testing period. Prior to each testing round, the experimenter evenly spaced 25 (size and color) marbles across the bedding surface. 16 mice were examined in separate cages during each testing round. After 30-minutes, each mouse was returned to its home cage and all marbles 2/3 or more covered by bedding were scored as buried by an experimenter blind to genotype.

*Acoustic startle and prepulse inhibition of the startle response*.

The apparatus consisted of twelve acoustic startle chambers for mice (SR-LAB, San Diego Instruments, San Diego, CA, USA). During the testing session animals were placed into clear Plexiglas cylindrical enclosure which rested on a platform in the center of the chamber. A piezoaccelerometer was connected to the platform to detect any vibrations caused by the animal’s activity in the enclosure. Startle responses to auditory stimuli were measured by amplitude of a whole body flinch. These responses were captured and digitized, and then sent to the computer for analysis.

Test session started when animals were individually placed into enclosures inside the sound-attenuated startle chambers and acclimated for 3 minutes. PPI was assessed in a test session lasting for 15 min, in which the subjects were presented with a series of discrete trials comprising a mixture of six types of trials: startle pulse (120 dB, for 40 ms), no stimulus (70 dB background noise), or prepulse stimulus of one of four sound levels (74, 78, 82 or 90 dB) for 20 ms, followed 100 ms later by an acoustic startle (120 dB for 40 ms). A total of 6 trials under each condition were delivered in a random sequence and all trials were separated by a variable inter-trial interval of 10–30 s. Max response to the stimulus (V max) was averaged for each trial type. In the startle alone trials, the basic auditory startle (startle response) was measured. In “no stimulus” trials a baseline measure was taken to assess movement in the enclosure under no stimulation. Animals with an average startle response value equal to or below 100 were excluded from PPI analysis as this value is similar to that obtained without stimulation and suggestive of no response to the startle pulse.

*Tail Suspension test*

The tail suspension test for depression-related behavior was conducted using 8 chambers from Med Associates (PHM-300TSS Mouse Tail Suspension System, Med Associates, Georgia, VT). The test cubicle is made of ½” white PVC with inside dimensions of 13” x 13” x 12.5”. The mouse was securely fastened with medical adhesive tape by the tip (~1.0-1.5 cm) of the tail to a metal hanger and suspended above the floor in a visually isolated cubicle. Mobility was recorded by a precision linear load cell, load cell amplifier, and filter with the gain set at 4. Immobility was defined as the area under the curve using an immobility threshold of 2. The total duration of immobility over a single 6-min session was recorded for each mouse. The test was repeated for three days. Animals that climbed their tails during testing on any of the test days were excluded from data analysis.
