# Supplementary figures and images for: Loss of the Putative Catalytic Domain of HDAC4 Leads to Reduced Thermal Nociception and Seizures while Allowing Normal Bone Development
Source: PLoS One. 2009 Aug 12;4(8):e6612. doi: 10.1371/journal.pone.0006612 (PMC2720538; doi:10.1371/journal.pone.0006612)

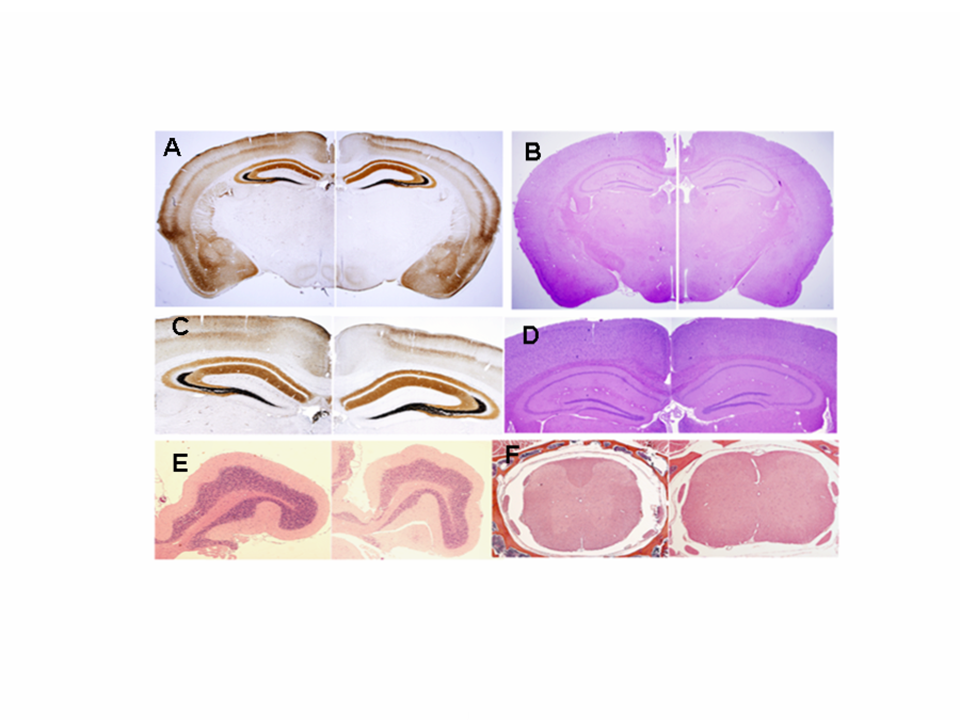

Supplement: Figure S1 — Histology of Brain: in each section left half is from WT and right half is from HDAC4ΔC mice. A and C is Timm staining of a coronal section through the hippocampus of a WT and an HDAC4ΔC mice. B, D, E and F is H&E staining of sections of a WT and a HDAC4ΔC mice. B & D shows the hippocampus is similar in WT and a HDAC4ΔC mice, E shows the cerebellar flocculus and F compares the spinal cord section of a WT and a HDAC4ΔC mice. (0.96 MB TIF) [file pone.0006612.s002.tif]

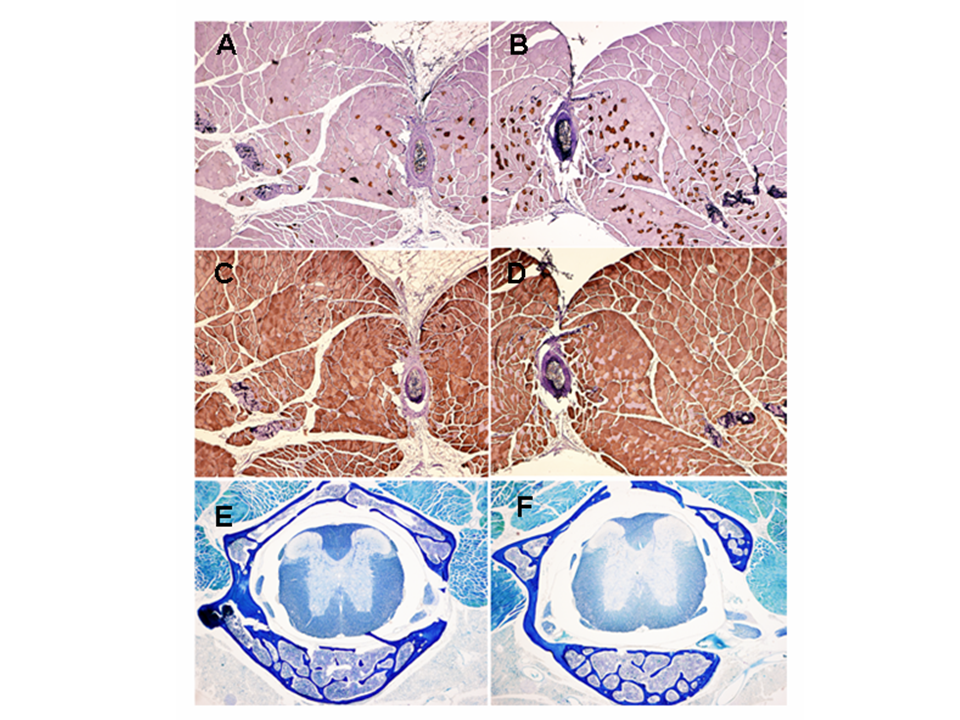

Supplement: Figure S2 — Histology of vertebral muscles and bone; A, C & E is WT; B, D & F is HDAC4ΔC mice. A&B shows IHC for MHC slow chain, C&D is IHC for MHC fast chain in lumbar vertebral muscles. E&F shows luxol fast blue stained lumbar vertebrae and spinal cord. (1.55 MB TIF) [file pone.0006612.s003.tif]
